# Factors contributing to persistent shoulder pain after arthroscopic rotator cuff repair: Protocol for a scoping review

**DOI:** 10.12688/f1000research.156193.1

**Published:** 2024-10-15

**Authors:** Anupama Prabhu B, G Arun Maiya, Vivek Pandey, Kiran K V Acharya, Prabu Raja G, James Elliott M, Mira Meeus

**Affiliations:** 1 Department of Physiotherapy, Manipal College of Health Professions, Manipal Academy of Higher Education, Manipal, Karnataka, 576104, India; 2 Department of Orthopedics, Kasturba Medical College, Manipal Academy of Higher Education, Manipal, Karnataka, 576104, India; 3 Department of Exercise and Sports Sciences, Manipal College of Health Professions, Manipal Academy of Higher Education, Manipal, Karnataka, 576104, India; 4 The University of Sydney, Sydney School of Health Sciences, Camperdown, New South Wales, 2050, India; 5 The Kolling Institute, St Leonards, Northern Sydney Precinct, Sydney, New South Wales, 2065, Australia; 6 University of Antwerp, Pain in Motion, Antwerp, Antwerp Province, 2000, Belgium; 7 Department of Rehabilitation sciences and Physiotherapy (REVAKI), MOVANT Research Group, University of Antwerp, Antwerp, Antwerp Province, 2000, Belgium

**Keywords:** Chronic pain, Predictors, Shoulder, Factor, Arthroscopy, Risk factor

## Abstract

**Introduction:**

Rotator cuff (RC) tears are the most common and disabling musculoskeletal ailments among patients with shoulder pain. Although most individuals show improvement in function and pain following arthroscopic rotator cuff repair (ARCR), a subgroup of patients continue to suffer from persistent shoulder pain following the surgical procedure. Identifying these factors is important in planning preoperative management to improve patient outcomes.

**Objective:**

This scoping review aims to identify biological factors, psychological factors, and social determinants of health contributing to the development of persistent pain in individuals after the ARCR procedure.

**Inclusion criteria:**

All prospective and retrospective longitudinal studies reporting the risk factors contributing to persistent pain three months or longer after the ARCR surgery will be considered for this scoping review.

**Methods:**

Our review will adhere to the Joanna Briggs Institute (JBI) scoping review methodology. Four electronic databases PubMed, CINAHL, Embase, and Scopus will be searched for studies in the English language. Additional studies can be found by conducting a citation analysis of the included studies. Title and abstract screening will be performed by two independent reviewers following the inclusion criteria, a third reviewer will be consulted about any differences. Next, full-text screening will be conducted, and the remaining search results will be reviewed to extract data, as well as to synthesize findings from all research. An overview of findings will be depicted in tabular format accompanied with a narrative summary of various factors contributing to persistent pain.

## Introduction

Shoulder pain ranks third among common, but enigmatic, musculoskeletal disorders, prompting patients to visit a wide variety of healthcare practitioners. ^1^ The general population’s prevalence of shoulder pain varies greatly, averaging 16% (range: 0.67 to 55.2%). According to the global burden of shoulder pain survey, the incidence of shoulder discomfort ranges from 7.7 to 62 per 1000 persons per year (median 37.8). ^2^ Accounting for more than half of the shoulder issues addressed by healthcare providers, RC disorders contribute significantly to shoulder pain and dysfunction. ^3, 4^ RC disorders are inclusive of RC tendinopathies, calcified tendinopathies, and partial/full-thickness RC tears. ^5^ The shoulder joint is the most mobile in the body because of the RC muscles’ contribution to its static and dynamic stability. ^6^ Between 4% and 32% of patients with RC disorders present with symptoms such as pain, weakness, and limitations in daily activities, reflecting the demand placed on these muscles. ^7^ Shoulder pain can also result in lost productivity, absences from work, and early retirement in workers, especially those who are subjected to heavy physical demands or repetitive overhead activities. ^8^ A systematic review has outlined the challenges in decision-making for conservative or surgical management of RC tears. ^9^ The review findings suggest that earlier surgical intervention may be necessary in cases of weakness and significant functional disability. It also appears that worker’s compensation claims may negatively impact the treatment outcomes.

Over the last decade, the surgical management of RC tears has changed from open to mini-open to arthroscopic repair. ^10^ Currently, ARCR surgery is the most common treatment for RC tears. After ARCR , functional outcomes of traumatic and non-traumatic RC tears have shown similar and satisfactory results. ^11– 13^ Improved function, quality of life, range of motion, strength, and pain relief can be attained with the ARCR procedure. ^14, 15^ The incidence of ARCR has seen a linear increase in various developed ^16^ and developing ^17^ countries and this rate is expected to rise with an increase in the aging population. The overall cost of ARCR surgery for the National Health Service in England exceeded £60 million, whilst the anticipated yearly cost of repair in the United States is said to be between US$1.2 and 1.6 billion. ^18^ Furthermore, there has been a prominent change in the performance of ARCR procedures at ambulatory surgery centers. These healthcare facilities are known for discharging patients on the same day they undergo surgery. ^19^

While several studies have reported positive outcomes on early postoperative pain control, a subgroup of patients report persistent shoulder pain of varying severity and duration following ARCR. ^20– 22^ Persistent post-surgical pain is defined by the International Classification of Diseases – 11 as pain that develops or gets worse following surgery or tissue damage and lasts for at least three months after the initial occurrence, beyond the healing phase. ^23^ The transition from postoperative acute pain to persistent post-surgical pain is a complex and multifactorial phenomenon. ^24^ Persistent postoperative pain is largely underdiagnosed and frequently inadequately managed. ^25^ Studies have reported reduced shoulder range of motion, strength deficits, and significantly lower functional scores in patients with persistent shoulder pain following the ARCR procedure. ^26^ Orthopedic surgeries in general are related to an increased risk of experiencing moderate to severe persistent pain at one-year follow-up when compared to other surgical procedures. ^27^ This could be due to the presence of pre-existing nociplastic pain and central sensitivity changes before the surgical intervention in a variety of orthopedic conditions. ^28, 29^ Research supporting the role of psychological variables in chronic shoulder pain is emerging. Preoperative psychological disorders such as depression and anxiety, as well as behaviors such as pain catastrophizing, kinesiophobia, and psychological discomfort, may cause patients to experience increased shoulder pain. ^30^

The degree of persistent postoperative pain beyond three months and the patterns of pain at the follow-up period are unknown. Numerous factors including fatty infiltration of the RC musculature, tear size, and diabetes mellitus are known to predict poor outcomes. ^31^ In addition, factors like opioid consumption, psychological and socioenvironmental, and acute postoperative conditions help explain the occurrence of persistent pain following ARCR. ^32^ Orthopedic surgeons and physical therapists may be better able to predict results and comprehend this patient population if they are aware of factors contributing to persistent pain following ARCR. Pre-operative pain control could be a preventive measure towards reducing the incidence and development of persistent postoperative pain. ^33^ It would be beneficial to identify patients at higher risk of developing persistent pain before the surgical procedure or during initial rehabilitation. This would enable targeted preoperative or perioperative interventions to better manage the transition to persistent postoperative pain. These targeted interventions could potentially be incorporated into a comprehensive care plan for patients undergoing ARCR. Numerous trials have been undertaken to assess the effectiveness of postoperative pharmacological therapies in reducing acute pain ^34, 35^ but no comprehensive evaluation has yet been conducted to assess factors contributing to persistent pain after ARCR.

### Review questions

This scoping review aims to identify factors reported in the literature that contribute to the development of persistent pain in patients following the ARCR procedure. The review will specifically aim to address the following questions.

i) Which pre-operative, intraoperative, and post-operative factors contribute to persistent pain of the shoulder in patients undergoing ARCR?
ii) Which biological factors, psychological factors, and social determinants of health contribute to persistent pain at three months or longer after the ARCR surgery in patients with RC tears?

## Methods

This protocol has been developed based on JBI methodology for scoping reviews ^36^ and will be in line with the Preferred Reporting Items for Systematic Reviews and Meta-Analyses extension for Scoping Reviews (PRISMA-ScR). ^37^ Details of this review project are available on Figshare: 10.6084/m9.figshare.26885746. ^38^

### Eligibility criteria

All prospective and retrospective longitudinal studies that include participants beyond 18 years of age, diagnosed with partial/full thickness (>5 cm) traumatic or degenerative RC tears undergoing primary ARCR where concomitant procedures like acromioplasty, subacromial decompression, biceps tenotomy, distal clavicle excision, and labral debridement, conducted with ARCR will be included. These concomitant procedures are often performed with ARCR to address additional aspects of shoulder joint pathology. Studies that have a combination of other orthopedic procedures will be included if separate data is available for the ARCR participants. Studies on massive tears (>5 cm in diameter or the detachment of two or more tendons) having a higher rate of recurrent tears and requiring complex surgical procedures like patch augmentation, muscle-tendon transfer, or reverse total shoulder arthroplasty will be excluded. ^39^ Revision repairs will, even so, be excluded as this will interfere with our data on primary ARCR.

The concept underlying this scoping review is the biological factors e.g. the size of tendon tears, tendon retraction, etc., and the psychological factors e.g. depression, catastrophizing, anxiety, etc., and social determinants of health e.g. gender, race, occupation, etc., related to persistent pain following ARCR surgery. For a study to be considered for inclusion in the scoping review the intensity of pain should be assessed on a reliable and valid unidimensional or multidimensional pain scale at any time point beyond three months following ARCR, identifying at least one pre-operative, intraoperative, or postoperative factor, and the relationship between the factor(s) and ongoing pain. Pharmacological and non-pharmacological studies providing interventions for pain relief will not be considered in this scoping review. Trials with a primary objective towards the efficacy of intervention rather than the impact of risk factors on pain outcome at follow-up will be excluded. The scoping review will encompass studies carried out in global hospitals and primary healthcare settings, considering evidence from any geographical location.

### Search strategy

The search strategy will be designed to find published original research, literature reviews, and written works. An initial limited search on PubMed was conducted to identify articles in this area. A thorough search method was developed using the terms present in the titles and abstracts of pertinent papers, along with the index terms and keywords utilized to describe the articles. An example of a PubMed search is provided in the extended data availability. ^40^ Title and abstract screening were performed to identify articles on persistent pain in patients who have undergone ARCR surgery. The search strategy will be adapted for every database that is included. Search will be conducted in electronic databases PubMed, CINAHL, Embase, and Scopus for further information. Articles published in the English language and full text available will be included. We will search the reference lists of all the included studies for any additional potentially eligible studies. Every study included needs to be published in a peer-reviewed academic publication. No date limitations will be applied in the review.

### Study selection

Following the search, all references will be gathered and added to the Rayyan online platform for systematic reviews developed by the Qatar Computing Research Institute in Doha, all duplicate references will be eliminated. Title and abstract screening will be carried out by two separate reviewers for the scoping review, using the inclusion and exclusion criteria. Two reviewers will thoroughly evaluate the complete content of possible eligible references in a second phase based on the eligibility criteria. Any disagreements between the two assessors at any stage of the screening procedure shall be resolved by discussion or by consulting a third assessor. A narrative scoping review summarizing the search findings, and the research inclusion procedure will be reported, along with a PRISMA flowchart.

### Data extraction

Two independent reviewers will use a standardized form to collect data from the final selected publications. The reviewers have developed a data extraction tool containing information that needs to be extracted from the articles. ^41^ The extracted information will include details about participants, concepts, study methodology, context, and important conclusions related to the review question. Details identifying the biological factors, psychological factors, or social determinants of health in the pre-operative, intraoperative, and/or post-operative period will be included. The data extraction tool will be piloted by two reviewers who will also make any necessary modifications. The final scoping review will include a detailed analysis of the modifications. Any disagreements between reviewers will be settled by discussion or by bringing in a third reviewer. We will contact the authors if there is any information missing or needed.

### Data analysis and presentation

Initially, a descriptive analysis of each study’s findings will be completed. The data extraction will be displayed in a tabular manner following the objectives of this scoping review. A preliminary version outlining the attributes of the studies included has been formulated for this scoping review and it may undergo further revision during the review. ^42^ The presentation of data will be categorized as described in the data extraction tool. At the individual predictor level, the consistency of reporting will be undertaken to determine the variables showing significant association with persistent pain. The review’s results will be summarized and reported to outline the factors contributing to persistent pain in patients receiving ARCR. ^43^ A narrative summary will be provided along with the tabulated results to explain how the findings correspond with the purpose and research inquiry of the scoping review. Research gaps will be summarized in a narrative report regarding factors associated with persistent pain after ARCR. We adhered to the Preferred Reporting Items for Systematic Reviews and Meta-Analysis Extension for Scoping Reviews (PRISMA-ScR) checklist to ensure standardized reporting. ^44^

## Disclosure statement

The authors have no conflict of interest to report.

## Ethics and consent

Ethics and consent are not applicable for this study.

## Data Availability

No data are associated with this article. Figshare: Extended Data File 1: Preprint, https://doi.org/10.6084/m9.figshare.26885746 ^38^ Figshare: Extended Data File 2: Search String, https://doi.org/10.6084/m9.figshare.26954635.v1 ^40^ Figshare: Extended Data File 3: Data extraction form, https://doi.org/10.6084/m9.figshare.26954698.v1 ^41^ Figshare: Extended Data Table 1: Characteristics of Included studies, https://doi.org/10.6084/m9.figshare.26954710.v1 ^42^ Figshare: Extended Data Table 2: Risk factors for persistent pain after arthroscopic rotator cuff repair, https://doi.org/10.6084/m9.figshare.26954833.v1 ^43^ Figshare: Extended Data File 4: PRISMA-ScR Checklist, https://doi.org/10.6084/m9.figshare.26936515.v1 ^44^ Data are available under the terms of the Creative Commons Zero “No rights reserved” data waiver (CC0 1.0 Public domain dedication).
