# Supplementary material for: Factors contributing to persistent shoulder pain after arthroscopic rotator cuff repair: Protocol for a scoping review
Source: F1000Res. 2024 Dec 6;13:1236. Originally published 2024 Oct 15. [Version 2] doi: 10.12688/f1000research.156193.2 (PMC11715645; doi:10.12688/f1000research.156193.2)
Supplement: Peer review history

# Reviewer report: Reviewer response for version 2

**DOI:** 10.5256/f1000research.175278.r346588

**Published:** 2024-12-14

**Authors:** Divya Bharatkumar Adhia

**Affiliations:** 1 University of Otago, Dunedin, New Zealand

The authors have addressed the comments I had and I have no further comments.

Is the study design appropriate for the research question?

Yes

Is the rationale for, and objectives of, the study clearly described?

Yes

Are sufficient details of the methods provided to allow replication by others?

Yes

Are the datasets clearly presented in a useable and accessible format?

Not applicable

Reviewer Expertise:

Chronic musculoskeletal pain

I confirm that I have read this submission and believe that I have an appropriate level of expertise to confirm that it is of an acceptable scientific standard.

# Reviewer report: Reviewer response for version 1

**DOI:** 10.5256/f1000research.171471.r336755

**Published:** 2024-11-25

**Authors:** Divya Bharatkumar Adhia

**Affiliations:** 1 University of Otago, Dunedin, New Zealand

The aims and methodology of this scoping review has been well presented.

 I only have one comment:

 The authors could consider conducting quality assessment for all the papers included in the review. This will add value to interpreting findings from the studies and evaluating any mixed results from multiple studies.

Is the study design appropriate for the research question?

Yes

Is the rationale for, and objectives of, the study clearly described?

Yes

Are sufficient details of the methods provided to allow replication by others?

Yes

Are the datasets clearly presented in a useable and accessible format?

Not applicable

Reviewer Expertise:

Chronic musculoskeletal pain

I confirm that I have read this submission and believe that I have an appropriate level of expertise to confirm that it is of an acceptable scientific standard, however I have significant reservations, as outlined above.

# Response

**Published:** 2024-12-03

**Authors:** Anupama Prabhu

**Affiliations:** Physiotherapy, Manipal Academy of Higher Education, Manipal, Karnataka, India

Thank you for your thoughtful suggestion regarding the inclusion of a quality assessment for the studies in our review.

 We agree that conducting a quality assessment would enhance the interpretation of findings and help evaluate any mixed results. Accordingly, we plan to assess the quality of all included articles using the Newcastle-Ottawa Quality Assessment Scale. This addition will ensure a more robust and comprehensive evaluation of the evidence.

# Reviewer report: Reviewer response for version 1

**DOI:** 10.5256/f1000research.171471.r332398

**Published:** 2024-10-25

**Authors:** Sanjay Tejraj Parmar

**Affiliations:** 1 SDM College of Physiotherapy, SDM University, Dharwad, India

1. Why are pharmacological and non pharmacological studies providing interventions for pain relief not considered in this review

 2. Please include all details of data extraction

 3. Process of resolving disagreements between reviewer should be clearly outlined

 4. Referencing needs correction

Is the study design appropriate for the research question?

Yes

Is the rationale for, and objectives of, the study clearly described?

Yes

Are sufficient details of the methods provided to allow replication by others?

Yes

Are the datasets clearly presented in a useable and accessible format?

Yes

Reviewer Expertise:

NA

I confirm that I have read this submission and believe that I have an appropriate level of expertise to confirm that it is of an acceptable scientific standard.

# Response

**Published:** 2024-12-03

**Authors:** Anupama Prabhu

**Affiliations:** Physiotherapy, Manipal Academy of Higher Education, Manipal, Karnataka, India

1. Thank you for your suggestion regarding including pharmacological and non-pharmacological interventions. Our primary aim is to identify the biological, psychological, and social factors that contribute to persistent pain lasting three months or longer after arthroscopic rotator cuff repair surgery in patients with rotator cuff tears. Including studies that aimed at comparing pharmacological and non-pharmacological interventions for pain relief falls outside the scope of our review as we do not aim at evaluating the effectiveness of specific interventions but rather to understand the multifaceted contributors to chronic post-surgical pain. Further, studies comparing different surgical procedures will also not be included for the same reason. This allows us to remain focused on understanding the chronic pain predictors, which could guide future research and interventions.
2. Thank you for your valuable feedback. We have revised the section on data extraction in our manuscript and included additional details to enhance clarity and transparency in our review protocol. We hope this addresses your concern effectively and improves the overall quality of our manuscript.
3. The process of resolving disagreements between reviewers has been outlined in our manuscript under the heading “study selection”.
4. Sorry for the oversight, references have been checked for appropriateness and revised where necessary.
